# A study on long-term trauma-related mental health outcomes among Kurdish survivors of chemical attacks

**DOI:** 10.3389/fpsyt.2025.1693072

**Published:** 2026-01-19

**Authors:** Ibrahim Mohammed, Hataw Ahmed, Bushra Hasin, Jan Kizilhan, Hemn Nariman, Salah Ahmed, Azad Qader, Sergi Papiol, Monika Rubekeil, Thomas Schulze, Urs Heilbronner, Martin Hautzinger

**Affiliations:** 1 Department of Psychology, Eberhard Karls University of Tübingen, Tübingen, Germany; 2 Department of Psychology, University of Sulaimani College of Medicine, Sulemani, Iraq; 3 Institute for Transcultural Health Science-Institute for Transcultural Health, Duale Hochschule Baden-Württemberg (DHBW) Stuttgart Research, Stuttgart, Germany; 4 Department of Pharmacy, Qaiwan International University, Sulaymaniyah, Iraq; 5 Jiyan Foundation For Human Rights, Erbil, Iraq; 6 Institute of Psychiatric Phenomics and Genomics (IPPG), Ludwig-Maximilians-Universität München (LMU) University Hospital, LMU Munich, Munich, Germany; 7 Department of Psychiatry and Behavioral Sciences, State University of New York (SUNY) Upstate Medical University, Syracuse, NY, United States; 8 Department of Psychiatry and Behavioral Sciences, Johns Hopkins University School of Medicine, Baltimore, MD, United States

**Keywords:** PTSD, depression, mental health, chemical attacks, war

## Abstract

**Background:**

The Kurdistan region experienced a series of devastating events, such as the 1988 chemical attacks and the 2014 Yazidi genocide, which have had substantial impacts on the psychological and physical health of survivors.

**Objectives:**

This study evaluates the long-term mental health of individuals who were exposed to chemical gas, with a focus on the prevalence and severity of psychological symptoms and their associations with sociodemographic, clinical, and trauma-related factors.

**Methods:**

A total of 534 participants were recruited (300 women and 234 men). Data collection was completed in 7 months, from March to September 2023. All participants completed validated psychological assessments, including the PTSD Checklist for *DSM-5* (PCL-5), Patient Health Questionnaire-15 (PHQ-15), and Hopkins Symptom Checklist-25 (HSCL-25). Multivariate General Linear Modeling (GLM) was performed, adjusting for trauma exposure to simultaneously assess the effects of demographic and clinical variables on multiple symptom domains.

**Results:**

Most of the participants exhibited clinically significant symptoms, with 78.8% meeting the post-traumatic stress disorder (PTSD) threshold and 46.3% exhibiting comorbid symptoms across multiple domains. The GLM demonstrated that gender, trauma exposure, education level, and clinical factors were significantly associated with symptom severity across PTSD, somatic, and anxiety/depression symptoms (p < 0.001). The model explained up to 47% of variance in symptom outcomes. After adjusting for covariates, women showed higher symptom severity than men. Greater trauma exposure and lower education independently predicted increased symptoms.

**Conclusion:**

These findings highlight serious psychological and somatic effects among survivors and underscore the urgent need for targeted mental health interventions for those affected by chemical attacks, with particular attention to individuals with lower levels of education and socioeconomic status, while incorporating gender-sensitive approaches to address differential vulnerabilities.

## 1 Introduction

Kurdistan is the name given to a large region spanning parts of Syria, Turkey, Iran, and Iraq, which is home to the Kurdish people. The Kurds represent the fourth-largest population, following Arabs, Turks, and Persians, in the Middle East; their population is estimated to range from 32 to 34 million people (1–6).

The Kurdistan Region in Iraq has witnessed several large-scale atrocities, including the Anfal operations in 1988, during which approximately 182,000 Kurdish people were killed and buried in mass graves. During this time, millions of civilians were displaced, and tens of thousands were transported to concentration camps. Thousands disappeared, and many villages were completely destroyed (7, 8). The seizure of Kurdish property in Iraq and mass killings have been prevalent since what is now called the Kurdish genocide of 1963, characterized by the burning of Kurdish villages, chemical warfare, ground attacks, and massive aerial bombardment (9).

In 1988, the city of Halabja suffered one of the most significant chemical attacks in the history of Kurdish genocide (10). The Iraqi regime used nerve agents, cyanide, and mustard gas. Officially, approximately 5,000 people from Halabja are estimated to have died in the attack, and thousands continue to suffer from its long-term consequences. The Ministry of Martyrs and Anfal Affairs in the Kurdistan Regional Government (KRG) reported that approximately 6,000 victims have survived the attack, many of whom still live with ongoing health issues (11).

Research on related psychological and physiological influences of exposure to chemical gas has remained limited over the years. Nevertheless, some studies are now beginning to show the far-reaching consequences of this atrocity on its victims. Survivors report high rates of post-traumatic stress disorder (PTSD), sleep problems, depression, social isolation, itching, and respiratory complications (11, 12).

Further research from the Kurdish context highlights the persistent impact of mass violence. For example, 87% of children who survived the Kurdish Anfal have been reported to have PTSD. The estimated prevalence rates of depression and PTSD in adults were 49% and 86.2%, respectively. Similarly, findings are reported among Iraqi Kurdish torture survivors, Syrian Kurdish refugees, and Yazidi genocide survivors, who often experience numerous overlapping conditions including PTSD, depression, and anxiety (13–16).

When compared with worldwide estimates, PTSD impacts approximately 3.9% of the general population during their lifetime, with prevalence increasing to approximately 48% in trauma-exposed populations (17, 18). These findings underscore the extraordinary mental health consequences of conflict-related trauma. Meta-analytic evidence further shows that over one-quarter of adult war survivors suffer from PTSD, and trauma exposure is also associated with depression, anxiety, and somatic symptoms like headaches, fatigue, and respiratory problems. Additionally, longitudinal studies among survivors of chemical weapon exposure show persistent psychiatric symptoms and physical comorbidities decades after exposure, highlighting the chronicity and complexity of trauma effects among survivors (19–21). Collectively, studies have demonstrated that exposure to severe trauma, such as chemical attacks, renders survivors vulnerable to long-lasting mental health disorders. These psychiatric conditions continue many years after conflict ends, particularly in low- and middle-income countries with limited access to healthcare. This chronicity underscores the important need to assess mental health outcomes long after the acute consequences of trauma, even decades following chemical warfare exposure (14, 22–25). Additionally, the psychological outcomes for chemical attack survivors cannot be limited to a single event. Rather, the chemical attack comprised multiple, interconnected traumatic events, such as physical injury, witnessing death, deprivation, and severe loss, which together contribute significantly to long-term mental health consequences (26). This complexity is consistent with an ecological model of trauma, which underscores the combined impact of direct exposure to violence and ongoing environmental stressors in war-affected populations. Therefore, evaluating cumulative trauma through composite measures results in a more precise and nuanced understanding of PTSD symptomatology in such contexts (27, 28).

Most mental health studies currently available in this context focus on the psychological impacts of mass killing and utilize relatively simple or univariate statistical methods. While many have assessed mental health consequences among Kurdish survivors of genocide and chemical attacks, important gaps remain. Limited attention has been given to the comprehensive assessment of both somatic and psychological symptomatology, despite evidence that physical health consequences significantly impact survivors’ overall health. Additionally, many studies have been limited by small sample sizes and lack of culturally adapted psychometric validation of measurement instruments (11, 14, 29, 30). In contrast, the present study integrates multiple psychological scales alongside sociodemographic and clinical factors to provide a more rigorous and multidimensional understanding of survivors’ mental health. The early findings of this study highlight the urgent need for additional research and more robust measures to determine the full impact of such atrocities as genocide and chemical attacks. Further study should not only emphasize on the psychological impacts but also incorporate physiological factors to better understand the long-term consequences and mental health impacts of large-scale, traumatic, and life-threatening events.

This study has attempted to fill the identified gap by developing a multi-method assessment approach and applying it to a large sample of survivors of chemical attacks. A comprehensive assessment of mental health status has been conducted, which reviews issues such as depression, anxiety, somatic symptoms, and post-traumatic stress. This research then contributes to establishing a comprehensive framework of the long-term psychological and psychiatric consequences of such traumatic events on the survivors.

In this first report, our aim is to comprehensively characterize the long-term psychological impact among Kurdish survivors of chemical weapons exposure by examining the prevalence, severity, and comorbidity of PTSD, depression/anxiety, somatic symptoms, and perceived stress. Additionally, the study seeks to investigate demographic, clinical, and trauma-related factors influencing symptom severity, including exploration of potential moderating effects. Prior to these analyses, the psychometric properties of the assessment instruments were evaluated to ensure that all instruments are reliable and appropriate to measure psychological symptoms of the study population.

## 2 Materials and methods

### 2.1 Ethical approval

This study received ethical approval from the ethics committee of the University of Duhok on 3 October 2021 (Approval Number: 141). In addition, we received permission from the Halabja governorate in April 2022 (Reference Number: 10/158), including the General Directorates of Security and Police. We were given permission to contact survivors at the Halabja Hospital for treatment of chemical victims. All survivors contacted (see below) were fully informed about the research and were voluntarily recruited to the study. If they decided not to participate, they did not face any negative consequences. All participants were assured of total confidentiality, and each participant consented in written form before the interviewing process began. The authors assert that all procedures contributing to this work comply with the ethical standards of the relevant national and institutional committees on human experimentation and with the Helsinki Declaration of 1975, as revised in 2013.

### 2.2 Study setting, period, and design

The primary data collection took place in Halabja city (*n* = 373), Sulemani city (*n* = 54), the Khormal district (*n* = 44), the Serwan district (*n* = 41), and the Tawella district (*n* = 22). It extended over 7 months, from March 2023 to September 2023. The design was a cross-sectional observational study involving Kurdish survivors of chemical gas attacks. The majority of participants were recruited from Halabja city, as this was the site of the 1988 chemical attack and the location of the largest population of survivors. Many survivors had relocated to other cities and districts due to displacement; therefore, recruitment efforts were extended to these areas to capture the dispersed survivor population. The sample size distribution reflects the practical realities of survivor location and accessibility rather than strict proportional allocation, as detailed population data for survivors across all areas were unavailable.

#### 2.2.1 Data collection procedure

A total of 534 samples were collected, with an additional 145 subjects who refused to be interviewed or could not be contacted. Inclusion criteria required that participants (1) have confirmed exposure to chemical gas, (2) had given informed consent to participate, and (3) were physically and mentally capable of completing the assessments. The exclusion criteria included the inability to provide informed consent and severe illness precluding participation. Participants were primarily recruited as Kurdish survivors of the chemical gas attacks based on official identification by the Kurdistan Regional Government’s Ministry of Martyrs and Anfal Affairs. Many survivors are officially registered as injured by chemical gas exposure and receive monthly financial compensation, which served as one pathway for recruitment. Considering that not all survivors are compensated, additional participants were identified through local NGOs working with survivors, especially those without official compensation. To further expand the sample size, snowball sampling was employed, in which initial participants referred other eligible survivors to the study. For those recruited outside official registries, victimization status for participants recruited outside official registries was assessed during structured interviews using standardized trauma exposure questionnaires. Initially, project details were explained to potential participants via phone, which was followed by in-person visits to their homes upon agreement to participate. Participants were briefed extensively on the project’s objectives, procedures, and potential risks. Each assessment required approximately 60 to 90 min to complete all questionnaires and interviews.

#### 2.2.2 Sample size determination and power analysis

The sample size was calculated using Cochran’s formula for proportions (31).


$$n_{0}\;=\frac{Z^{2}\times p\times (1-p)}{e^{2}}$$

in which *n*_0_ is the required minimum sample size, *Z* = 1.96 is the *Z*-score corresponding to a 95% confidence level, *p* = 0.5 is the estimated proportion of the attribute in the population (chosen as 0.5 to maximize variability), and *e* = 0.05 is the target margin of error (5%). Considering these values, the minimum sample size needed was 384. In order to ensure adequate power and representativeness of the study findings, the sample size was increased to 534 in order to account for potentially non-response and refusals, which were estimated to be 28% based on recruitment challenges. Non-response rates in similar vulnerable populations reported to range from 20% to 40% (32, 33).

Furthermore, this sample provides sufficient statistical power for multivariate analyses using a General Linear Modeling (GLM) including 12 predictors. *Post-hoc* calculations using Cohen’s *f*² = 0.15 and alpha (*α*) = 0.05 yielded an estimated power of >0.99, confirming that the study is well powered to identify effects on psychiatric symptomatology.

### 2.3 Data collection instruments

#### 2.3.1 PTSD checklist for DSM-5

PTSD checklist for *DSM-5* (PCL-5) is a 20-item self-report scale assessing *DSM-5* PTSD symptoms over the past month. Items are rated on a five-point Likert scale (0 = not at all to 4 = extremely). Total symptom severity scores are calculated by summing item responses, with a cutoff score ≥23 indicating probable PTSD (34). The PCL-5 has been validated for use in Kurdish trauma-affected populations and is conventionally designed to assess symptom severity related to a single, identified traumatic event. However, given the participants’ exposure to multiple overlapping traumatic experiences during the chemical attacks, including injury, deprivation, loss of family members, respiratory distress, displacement, and witnessing death, it was necessary to adapt this approach. Participants were instructed to respond to the PCL-5 items with respect to their cumulative trauma experience throughout the chemical attack period. This adaptation is consistent with the ecological model of trauma, which recognizes that mental health outcomes arise from both direct violence exposure and ongoing contextual stressors. Existing psychometric and epidemiological evidence validates this composite trauma framework as an appropriate method for assessing PTSD symptoms in post-conflict populations (27, 28, 35).

#### 2.3.2 Hopkins Symptom Checklist-25

Hopkins Symptom Checklist-25 (HSCL-25) is a 25-item scale measuring symptoms of depression and anxiety on a four-point Likert scale (not at all to extremely). The first 10 items measure anxiety symptoms, and the remaining 15 items assess depression symptoms. Mean scores are used, with a recommended cutoff of 1.75 for clinically significant symptoms validated in prior studies with chemical attack survivors (36).

#### 2.3.3 Modified Patient Health Questionnaire-15 (13 items)

PHQ-15 consists of 15 items assessing somatic symptoms; items are rated on a three-point scale (0 = not bothered at all, 1 = bothered a little, and 2 = bothered a lot). Severity categories are defined by summed scores of ≥5 mild, ≥10 moderate, and ≥15 severe (37). Two items in PHQ15 were removed. Item PHQ4 (“menstrual cramps or other problem with your period”) was removed as this item is specific to women and thus resulted in 49.4% missing data. According to the Item Response Theory (IRT) analysis, which was performed using a Graded Response Model (GRM) in R (38), PHQ4 showed low discrimination (a1 = 0.653), low factor loading (0.358), and low corrected item–total correlation (0.285), indicating that it poorly differentiates between respondents and contributes little to the overall scale. Similarly, item PHQ11 (“pain and problems during sexual intercourse”) was eliminated because 34.8% of the data were missing, It also demonstrated low discrimination (a1 = 0.659), low factor loading (0.361), and a low corrected item–total correlation (0.274), suggesting limited relevance to the measured construct. Additionally, a previous study suggested that item 4 and item 11 should be removed because these items reduce the overall sensitivity of the scale (39). To maintain comparability with the standard PHQ-15 scoring and cutoff thresholds (≥5, ≥10, and ≥15 for mild, moderate, and severe symptoms), we prorated total scores. In particular, the sum of the 13 items was multiplied by 15 (original item count) and divided by 13 (number of items retained), producing a prorated total score aligned with the original 15-item scale range (0–30). This prorated score was then used for all analyses and cutoff interpretations, preserving valid comparison with established severity categories.

#### 2.3.4 Perceived Stress Scale-14

Perceived Stress Scale-14 (PSS-14) is a 14-item scale measuring subjective stress levels on a five-point Likert scale from (0 = never to 4 = very often) over the past month. Total scores are calculated by summing item responses, with higher scores reflecting greater perceived stress (40).

#### 2.3.5 War and Adversity Exposure Checklist-26

War and Adversity Exposure Checklist-26 (WAEC-26) combined with 11 supplementary context-specific questions related to chemical attack events for measuring traumatic events. Each item is dichotomously scored (yes/no). The total trauma exposure score was calculated by summing all endorsed items, including the 11 supplementary questions (34).

Scale thresholds were determined through consultation with individual experts and organizations experienced in working with trauma survivors in the Kurdistan region, as well as through review of the existing literature (30, 34, 36, 41, 42).

Demographic information, including age, gender, education, marital status, occupation, and income, as well as chronic disease status, family history of mental illness, prior psychiatric diagnoses, and current psychotropic medication were collected through structured interviews. Chronic disease was defined as the presence of diagnosed, long-term medical conditions requiring continuous management, such as cardiovascular disease and diabetes. Family history of mental illness referred to any first-degree relatives with a diagnosed psychiatric disorder. Information on participants’ prior psychiatric diagnoses and current psychotropic medication use was obtained to determine whether participants had previously visited a psychiatrist; received formal diagnoses such as depression, PTSD, and anxiety; or were currently taking psychotropic medications. Although formal psychiatric diagnosis based on standardized criteria such as the *Diagnostic and Statistical Manual of Mental Disorders, Fifth Edition* (*DSM-5*) and the International Classification of Diseases, 11th Revision (ICD-11) is the clinical gold standard, clinical history obtained via self-report or clinical interviews provides important pragmatic context in epidemiological research where structured clinical interviews may not always be feasible. Medication use was coded as a binary variable (yes/no) in analyses due to small numbers in specific medication categories. These clinical variables were included as covariates in all relevant analyses to control for their potential confounding effects on symptom severity.

All scales were translated into Kurdish/Sorani by two independent, qualified translators who were proficient in both English and Kurdish. The Kurdish versions were back-translated into English by new translators to verify the quality of the back translation, i.e., to ensure linguistic equivalence and conceptual similarity. Discrepancies between the back-translated versions and the original English questionnaires were dealt with in the revisions.

### 2.4 Data quality control

Data quality control included examination of frequency distributions for categorical variables and descriptive statistics (mean, median, standard deviation, skewness, and kurtosis) for continuous and ordinal variables, including individual items of the key psychometric scales (HSCL-25, PCL-5, PHQ-15, and PSS-14) to assess response distributions and identify anomalies. Continuous demographic and clinical variables were similarly inspected. We identified and removed duplicate cases using unique participant identifiers and verified data variable types and coding consistency. Internal consistency reliability of the scales was assessed with Cronbach’s alpha (*α*) and Kuder–Richardson Formula 20 (KR-20) as appropriate.

### 2.5 Statistical analysis

Both R version 4.4.2 and IBM SPSS Statistics version 29 were utilized for statistical analysis. Descriptive statistics, Cronbach’s *α*, and KR-20 were performed to measure the internal consistency. IRT was used specifically for the PHQ-15 to assess individual item performance and guide the removal of poorly performing items. The GRM was employed because it is appropriate for analyzing Likert-scale items with ordered response categories, allowing estimation of item discrimination and difficulty parameters. Pearson correlation was conducted to assess relationships among psychological variables. Multivariate analyses were to evaluate the effects of demographic and clinical variables on mental health outcomes. The GLM was run in R using the manova() function from the base stats package, with Pillai’s Trace as the test statistic due to its robustness against assumption violations such as heterogeneity of covariance matrices, which was assessed using Box’s *M* test [boxM() function from the heplots package]. Homogeneity of variances for individual outcomes was evaluated using Levene’s test [leveneTest() from the car package]. To ensure adequate statistical power for identifying medium effect sizes in models with 12 predictors, a *post-hoc* power analysis was conducted using the pwr package.

## 3 Results

### 3.1 Scales reliability

Data quality checks revealed no significant outliers or anomalies in item responses across all scales and variables. All translated and self-developed measures showed good to excellent internal consistency. The PCL-5 had *α* = 0.885, the HSCL-25 scale reached *α* = 0.936, the PHQ-13 had *α* = 0.868, the PSS-14 had *α* = 0.875, and the WAEC-26, when combined with the 11 additional supplementary questions, also demonstrated good internal consistency (KR-20 = 0.77), assessed using KR-20 since the scale was dichotomous with (yes/no) response options.

### 3.2 Sample characteristics and demographic variables

A total of 534 survivors of chemical gas exposure participated in the study. Sample characteristics are summarized in **Table 1**. The sample was 56.2% female, with a mean age of 53.6 years. Most participants had elementary education 44% and low income. The majority were married and homemakers or government employees, with a high rate of chronic disease.

**Table 1:** Demographic and clinical characteristics of the sample (*N* = 534).

Demographic variables	Frequency *n* (%) or mean (SD)
Sample	534 (100%)
Female	300 (56.2%)
Age, mean (SD)	53.6 (11.16)
Income in US$, mean (SD)	419.26 (269.49)
Level of education	
No formal education	122 (22.8%)
Elementary school	235 (44.0%)
Secondary school	77 (14.4%)
Institution or university	100 (18.7%)
Work status	
Unemployed	24 (4.5%)
Homeworker	228 (42.7%)
Governmental employee	158 (29.6%)
Self-employee	66 (12.4%)
Unable to work	7 (1.3%)
Retired	51 (9.6%)
Marital status	
Single	48 (9.0%)
Married	444 (83.1%)
Divorced	13 (2.4%)
Widow/Widower	29 (5.4%)
Number of children, mean (SD)	4.4 (2.6)
Chronic disease	
Having chronic disease	367 (68.7%)

#### 3.2.1 Symptom severity and clinical prevalence

**Figure 1:**
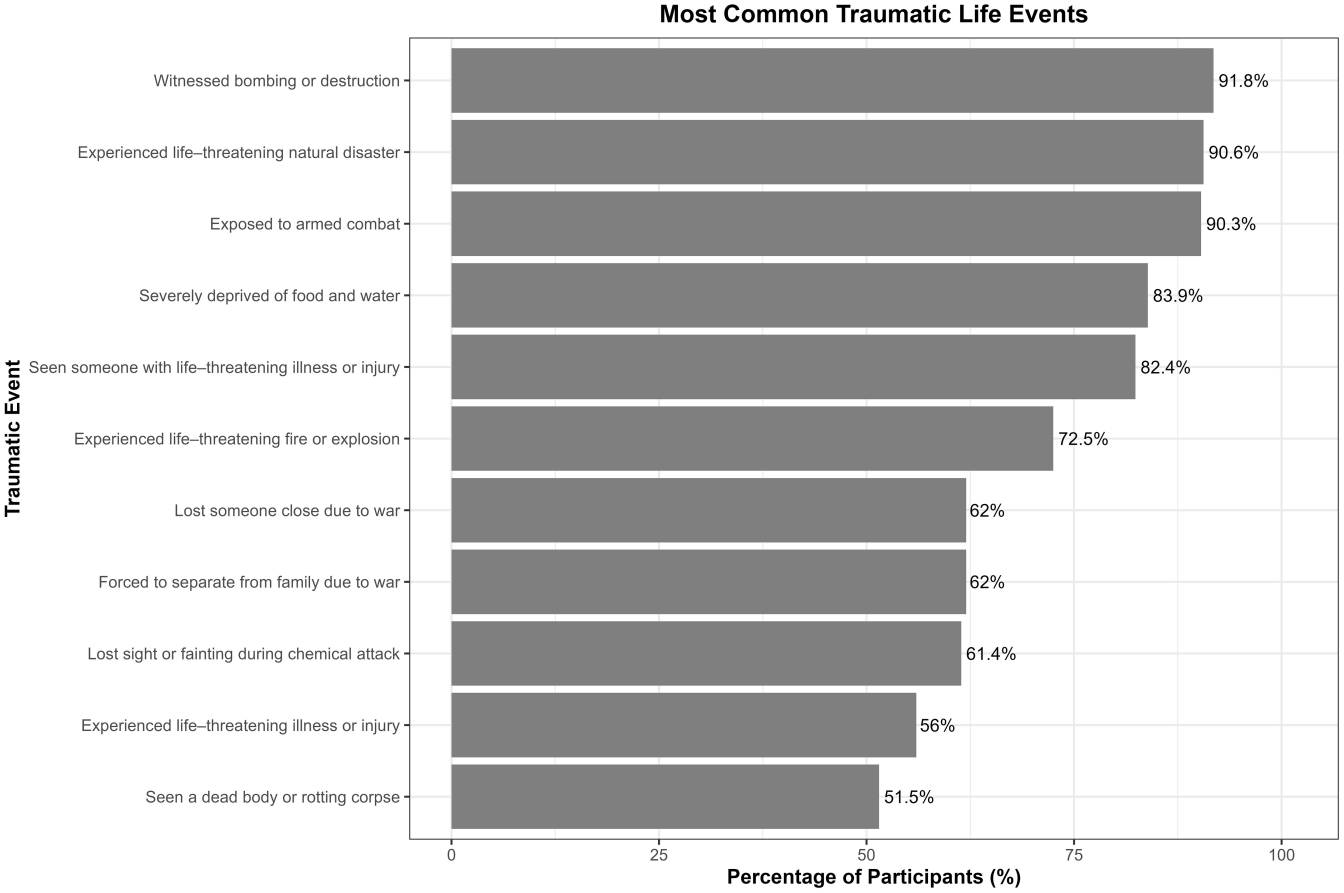
Common traumatic life events experienced by Kurdish survivors of chemical attacks.

**Figure 2:**
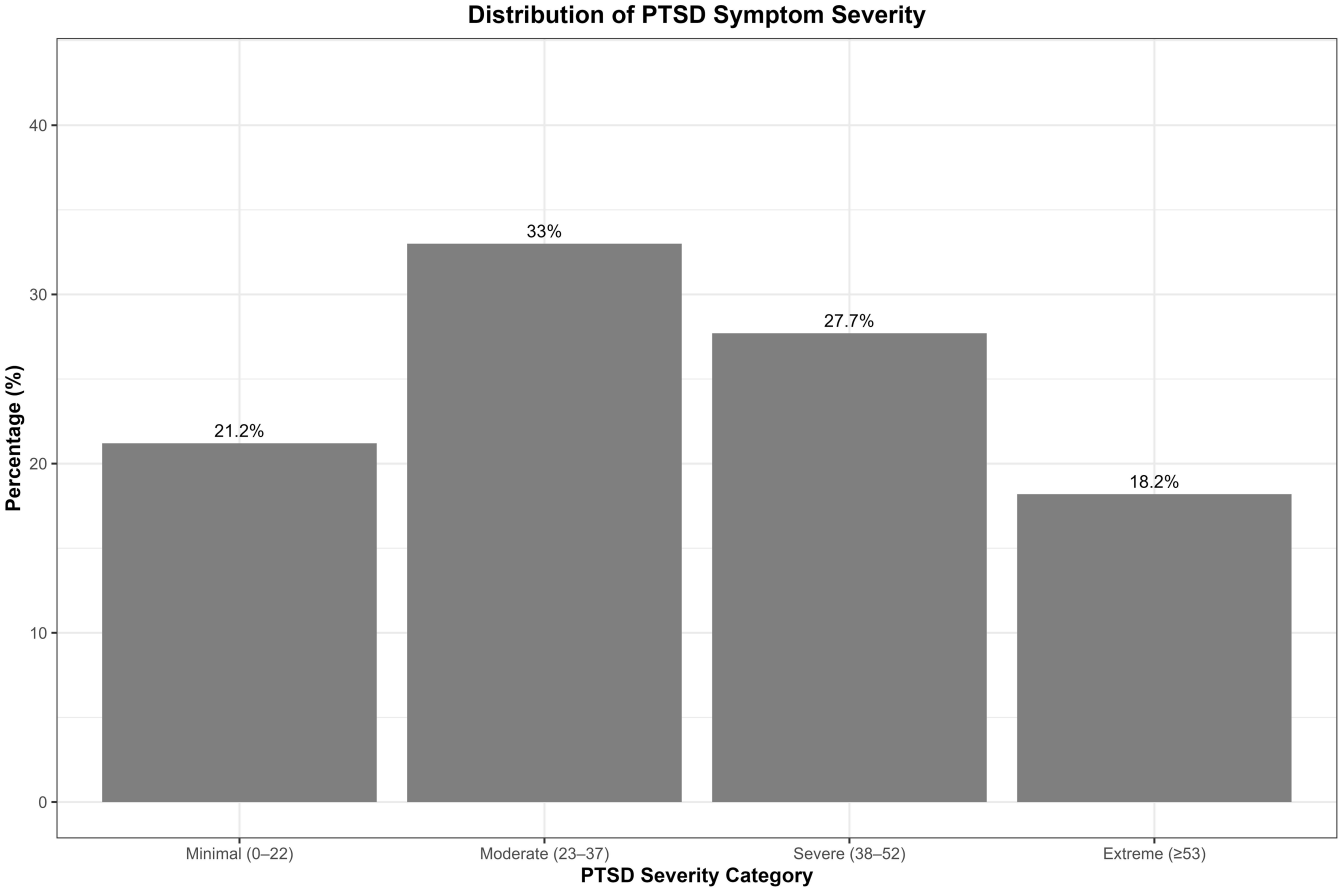
Distribution of PTSD symptom severity categories based on PCL-5 total scores. Categories were defined for descriptive purposes only. Bars represent the percentage of participants in each category; values on top of the bars show these percentages.

The mean (*M*) score and standard deviation (SD) for the PCL-5 were 36.14 and 16.05, respectively; PHQ-13: *M* = 15.72, SD = 7.33; HSCL25: *M* = 2.07, SD = 0.66; and PSS14: *M* = 24.30, SD = 11.14. Combined traumatic events (37 items) (*M* = 14.33, SD = 4.66) ranged from 2 to 30 traumatic events. The most frequently reported extreme symptoms for the PHQ-13 were “pain in your arms, legs, or joints” (74.7%), “feeling tired or having low energy” (66.1%), and “back pain” (64.6%). For the PCL-5, common symptoms were “repeated, disturbing, and unwanted memories” (38%), “feeling very upset when something reminded you of the stressful experience” (23.8%), and “trouble falling or staying asleep” (30.5%). Regarding HSCL-25, “feeling tense or keyed up” (26.4%) and “loss of sexual interest or pleasure” (26.8%) were the most frequently reported. The most frequently reported events were witnessing bombing or destruction (91.8%), experiencing life-threatening natural disasters (90.6%), exposure to armed combat (90.3%), and being severely deprived of food and water (83.9%). Less common events included seeing dead bodies and life-threatening illness or injury (**Figure 1**). The distribution of PCL-5 symptom severity categories is illustrated in **Figure 2**.

Related to clinically relevant cutoff scores, we found for PCL-5 that 78.8% of participants scored ≥23 on the PCL-5. For the HSCL-25, 65.2% had scored ≥1.75. For PHQ13, 10.1% of participants had minimal symptoms (score < 5), 12.9% had mild symptoms (≥5), 21.2% had moderate symptoms (≥10), and 55.8% had severe somatic symptoms (≥15).

#### 3.2.2 Symptom comorbidities

Comorbidities were common; 46.3% of participants met cutoff criteria for all three symptom domains (PTSD, somatic symptoms, and depression/anxiety); 65.2% of participants had PTSD and depression/anxiety symptoms, 51.5% had PTSD and somatic symptoms, while 47.8% had depression/anxiety and somatic symptom comorbidities.

#### 3.2.3 Psychotropic medication use and diagnostic profiles

**Figure 3:**
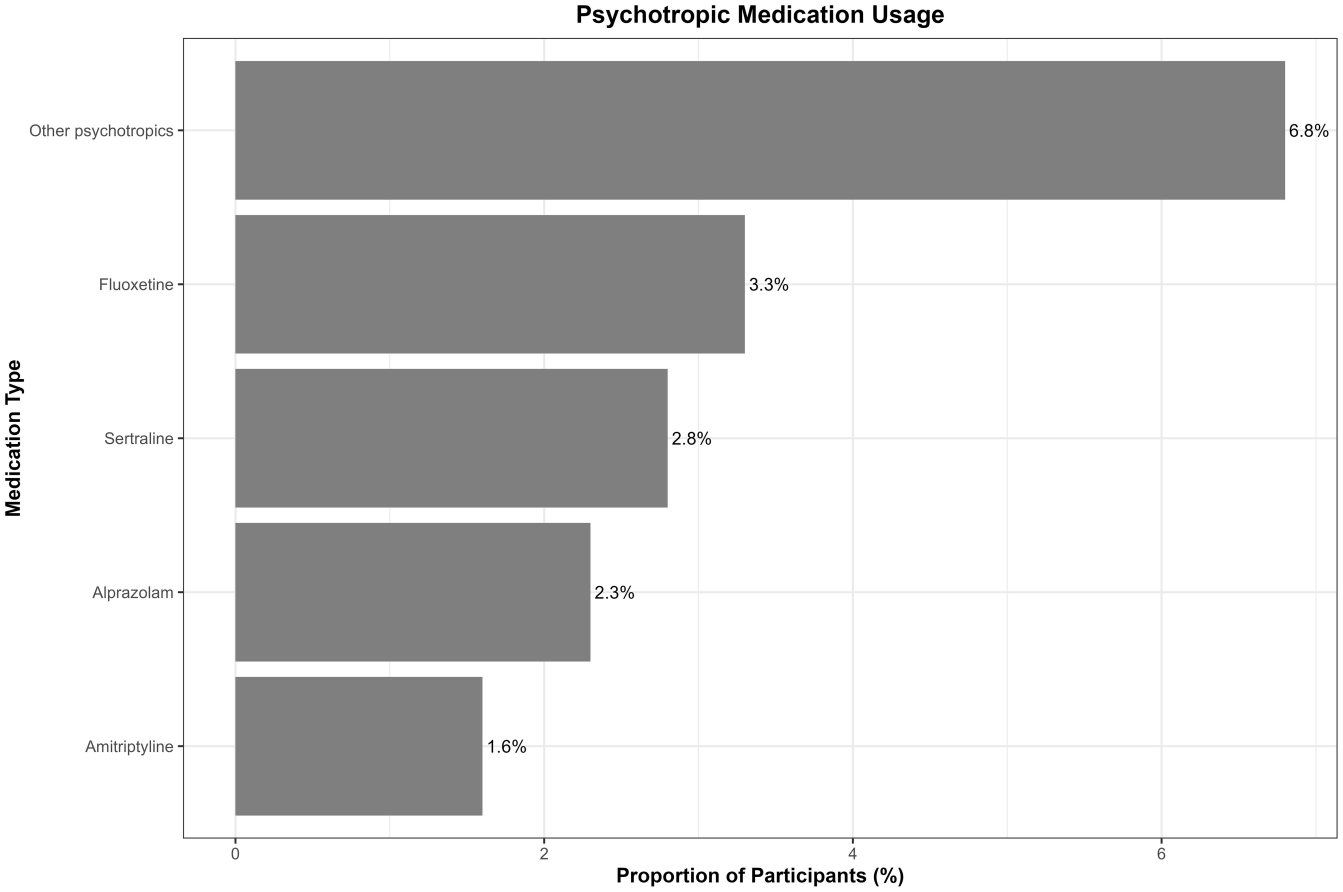
Psychotropic medication usage by type among study participants. Bar chart displays the proportion of participants prescribed each major psychotropic medication category.

**Figure 4:**
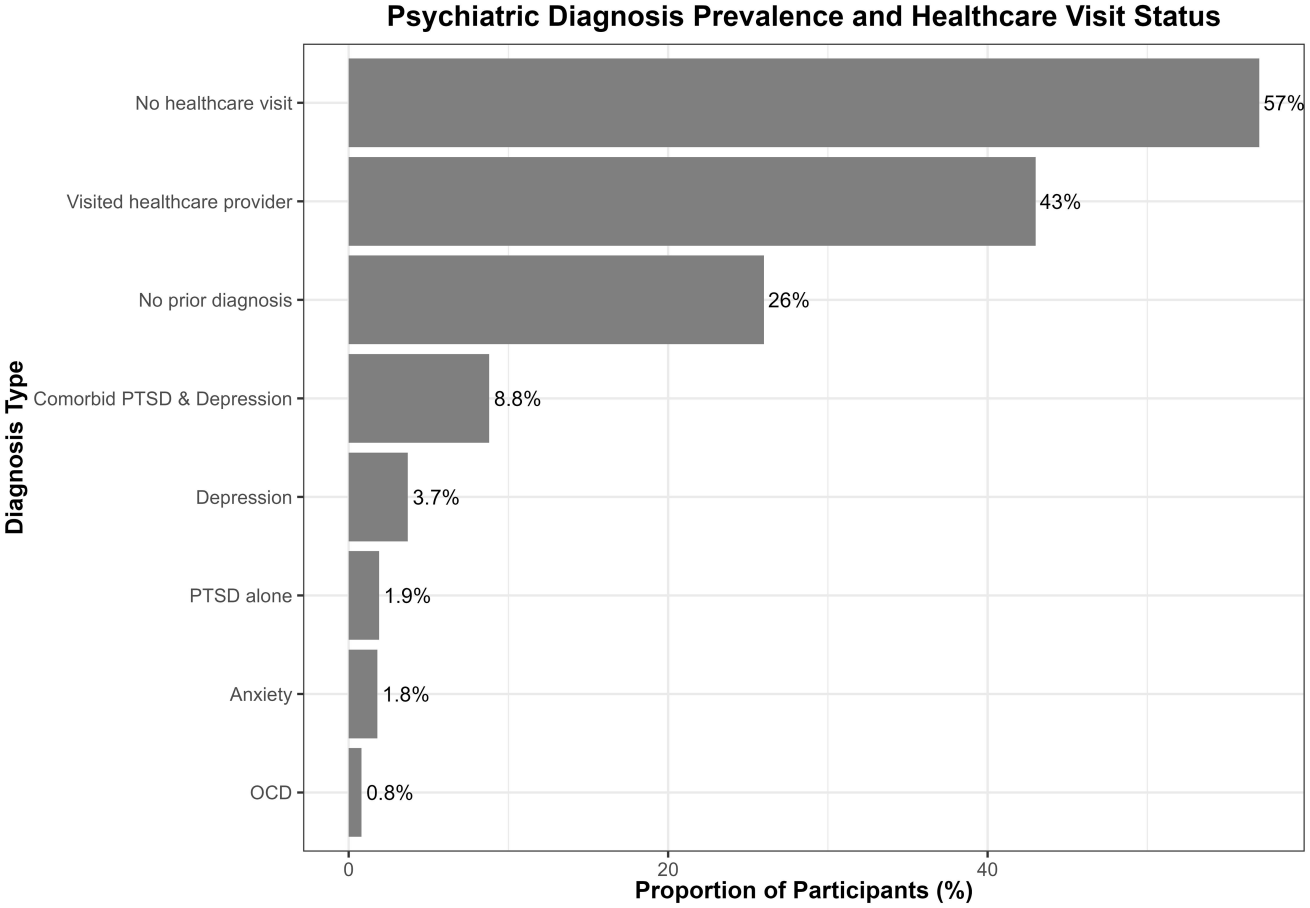
Prevalence of prior psychiatric diagnoses and healthcare professional visit status among participants.

Of the total participants, 16.8% reported current use of psychotropic medications. The other psychotropics had the highest proportion (6.4%), followed by fluoxetine (3.1%), sertraline (2.6%), alprazolam (2.2%), and amitriptyline (1.5%) (**Figure 3**). Approximately 26.0% reported no prior psychological diagnosis, while 8.8% reported comorbid PTSD and depression, with smaller proportions reporting PTSD alone (1.9%), depression (3.7%), anxiety (1.8%), and obsessive–compulsive disorder (OCD) (0.8%), while the majority (57%) did not visit a healthcare provider (**Figure 4**). The comorbid group consists of participants meeting criteria for both PTSD and depression, distinct from those with only one disorder.

### 3.3 Correlations

To explore the relationships among psychological symptoms, Pearson correlation analyses were performed. These revealed a significant positive correlation among all scales, *p* < 0.001. PCL-5 shows a strong positive correlation with HSCL-25 (*r* = 0.80) and somatic symptoms (PHQ-13, *r* = 0.69) and a moderate correlation with PSS-14 (*r* = 0.58), but a weaker positive correlation with the trauma checklist (*r* = 0.25). All correlations remained significant after Bonferroni correction (*p* < 0.0033) (**Table 2**).

**Table 2:** Pearson correlations among psychological symptom scales, presenting significant positive associations between the PCL-5, HSCL-25, PHQ-13, PSS-14, and the Trauma Event Checklist, all significant at *p* < 0.001.

Variable	PHQ-13	PCL-5	HSCL-25	PSS-14	Trauma Event Checklist-37
PHQ-13	1	0.696**	0.742**	0.506**	0.196**
PCL-5	0.696**	1	0.805**	0.583**	0.258**
HSCL-25	0.742**	0.805**	1	0.719**	0.235**
PSS-14	0.506**	0.583**	0.719**	1	0.158**
Trauma Event Checklist-37	0.196**	0.258**	0.235**	0.158**	1

**Indicates significance at *p* < 0.001 (two-tailed).

### 3.4 Multivariate general linear model analysis

GLM was performed to assess the effects of demographic, clinical, and treatment-related predictors on three related mental health outcomes: somatic symptoms (PHQ-13), PTSD symptoms (PCL-5), and anxiety/depression symptoms (HSCL-25). Assumption checks revealed a significant heterogeneity of covariance matrices across groups [Box’s *M*, *χ*²(42) = 65.84, *p* = 0.011]. Since Box’s *M* is sensitive to sample size and can detect minor differences, Pillai’s Trace was used for multivariate testing due to its robustness against violation of covariance homogeneity assumptions. Additionally, Levene’s tests for homogeneity of variance for each outcome were nonsignificant; PHQ-13 (*p* = 0.236), PCL-5 (*p* = 0.936), and HSCL-25 (*p* = 0.114), supporting the assumption of equal variances for the univariate analyses.

#### 3.4.1 Explained variance of the model

The multivariate model explained significant variance in symptom severity across the three outcomes, with adjusted *R*² values of approximately 0.47 for somatic symptoms, 0.35 for PTSD symptoms, and 0.43 for anxiety/depression symptoms.

#### 3.4.2 Multivariate effects

Significant main effects were observed for gender [Pillai’s Trace = 0.187, *F*(3,504) = 38.66, *p* < 0.001], education [Pillai’s Trace = 0.187, *F*(9,1518) = 11.21, *p* < 0.001], trauma exposure [Pillai’s Trace = 0.184, *F*(3,504) = 37.90, *p* < 0.001], family history of mental disorder [Pillai’s Trace = 0.038, *F*(3,504) = 6.69, *p* < 0.001], living location [Pillai’s Trace = 0.049, *F*(6,1010) = 4.25, *p* < 0.001], chronic disease [Pillai’s Trace = 0.023, *F*(3,504) = 3.99, *p* = 0.008], income [Pillai’s Trace = 0.205, *F*(3,504) = 43.41, *p* < 0.001], occupation [Pillai’s Trace = 0.056, *F*(15,1518) = 1.92, *p* = 0.018], psychotropic medication use [Pillai’s Trace = 0.120, *F*(3,504) = 22.87, *p* < 0.001], and prior diagnosis [Pillai’s Trace = 0.086, *F*(18,1518) = 2.48, *p* < 0.001]. Age, social status, and social support were not significant multivariately. The gender × education interaction was not significant [Pillai’s Trace = 0.028, *F*(9,1518) = 1.59, *p* = 0.11] (see **Supplementary Table S1**).

#### 3.4.3 Univariate effects and interaction details

Gender had significant main effects on all outcomes, somatic symptoms [*F*(1,506) = 112.64, *p* < 0.001, Partial eta squared effect size (partial *η*²) = 0.18; coefficient *β* = 0.037], PTSD symptoms [*F*(1,506) = 30.53, *p* < 0.001, partial *η*² = 0.06; *β* = 1.847], and depression/anxiety symptoms [*F*(1,506) = 54.95, *p* < 0.001, partial *η*² = 0.10; *β* = 0.034]; symptom severity was higher among women.

**Figure 5:**
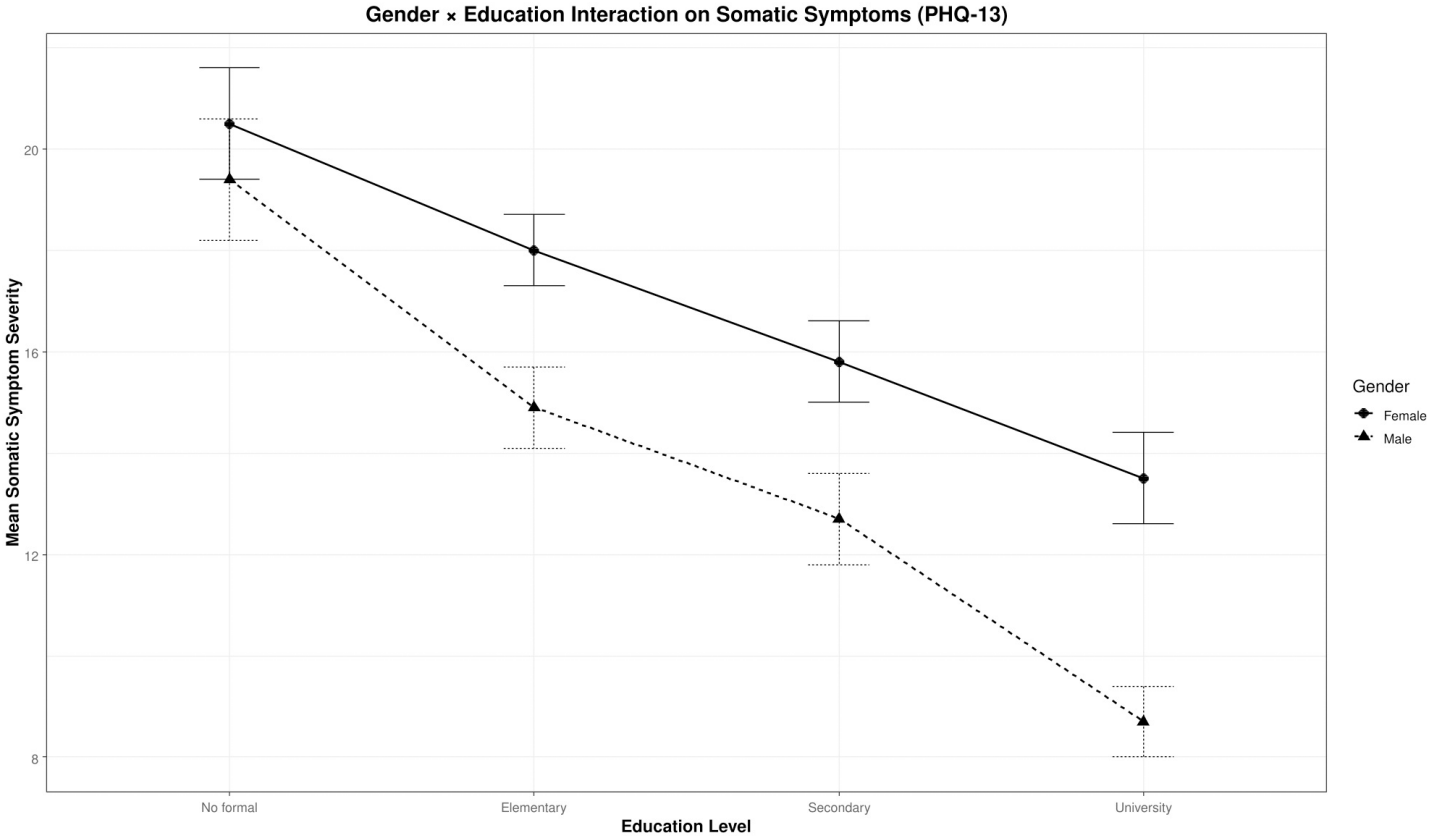
Interaction between gender and education on somatic symptoms (PHQ-13). Estimated marginal means of PHQ-13 somatic symptoms are plotted by education level for female participants (solid line) and male participants (dashed line).

Education showed significant main effects on all symptom domains, with the strongest effect on somatic symptoms [*F*(3,506) = 30.91, *p* < 0.001, partial *η*² = 0.15; *β* = −5.17 to −8.38 across levels]. Higher education levels were associated with lower symptom severity. The gender × education interaction was significant for somatic symptoms [*F*(3,506) = 4.55, *p* = 0.004, partial *η*² = 0.03; *β* = 2.84 to 5.99], revealing that higher education is a protective factor against somatic symptom severity in both genders, with a marked reduction observed among female participants (**Figure 5**).

Income was significantly associated with symptom severity across all outcomes: somatic symptoms [*F*(1,506) = 125.75, *p* < 0.001, partial *η*² = 0.46; *β* = −0.009], PTSD [*F*(1,506) = 51.46, *p* < 0.001, partial *η*² = 0.34; *β* = −0.012], and depression/anxiety symptoms [*F*(1,506) = 68.15, *p* < 0.001, partial *η*² = 0.57; *β* = −0.001], with higher income associated with fewer symptoms.

Trauma exposure was strongly associated with increased symptoms across somatic [*F*(1,506) = 84.58, *p* < 0.001, partial *η*² = 0.30; *β* = 0.34], PTSD [*F*(1,506) = 80.43, *p* < 0.001, partial *η*² = 0.38; *β* = 0.85], and depression/anxiety symptoms [*F*(1,506) = 83.82, *p* < 0.001, partial *η*² = 0.34; *β* = 0.031].

Family history of mental disorder significantly predicted depression/anxiety symptoms [*F*(1,506) = 10.98, *p* < 0.001, partial *η*² = 0.05; *β* = 0.089]. Living location was associated with somatic [*F*(2,506) = 8.82, *p* < 0.001, partial *η*² = 0.03; *β* = 2.54] and depression/anxiety symptoms [*F*(2,506) = 9.44, *p* < 0.001, partial *η*² = 0.04; *β* = 0.21], with urban residents reporting higher symptom severity compared to those living in peripheral areas or in villages. Chronic disease showed a modest association with somatic symptoms [*F*(1,506) = 9.57, *p* = 0.002, partial *η*² = 0.03; *β* = 1.07].

Occupation had a significant effect only on depression/anxiety symptoms across occupational groups [*F*(5,506) = 3.30, *p* = 0.006, partial *η*^2^ = 0.03], but showed no significant effect on PTSD and somatic symptoms.

Psychotropic medication use was associated with worse symptom severity across all outcomes, somatic symptoms [*F*(1,506) = 25.74, *p* < 0.001, partial *η*² = 0.05, *β* = 2.31], PTSD [*F*(1,506) = 42.89, *p* < 0.001, partial *η*² = 0.08, *β* = 6.93], and depression/anxiety symptoms [*F*(1,506) = 67.43, *p* < 0.001, partial *η*² = 0.12, *β* = 0.30].

Prior diagnosis was a significant predictor of PTSD [*F*(6,506) = 4.62, *p* < 0.001, partial *η*^2^ = 0.05, *β* = 10.11] and depression/anxiety symptoms [*F*(6,506) = 6.26, *p* < 0.001, partial *η*^2^ = 0.05, *β* = 0.53], but not somatic symptoms (*p* = 0.17).

Interactions between education × trauma exposure and income × trauma exposure were not significant, indicating that the effects of trauma exposure on symptoms do not differ across education or income levels in this sample (see **Supplementary Table S2** for details).

## 4 Discussion

To our knowledge, this is the first study conducted on the long-term psychological outcomes of a chemical weapons attack on a large community. We studied survivors of a chemical attack that occurred more than three decades ago to assess their current wellbeing and psychopathology. These findings fill a critical gap in the literature on prolonged trauma effects.

Most survivors met criteria for probable PTSD (78.8%) using a cutoff score of ≥23, which is lower than thresholds typically used in other populations (≥31–33) (43), possibly contributing to the high prevalence observed. Additionally, a significant proportion suffered from depression/anxiety and somatic symptoms.

These results show that extreme and chronic stress exposure has lasting negative effects on mental health, documenting the collective trauma experienced by populations exposed to genocidal attacks. The high rate of depression/anxiety and somatic symptoms reflects the psychopathological burden beyond PTSD and the need for effective interventions that target comorbidity of psychopathology.

These results are in line with previous findings that revealed a high prevalence of PTSD and depression among Anfal survivors and a high prevalence of PTSD among survivors of the Yazidi genocide. They also align with earlier research showing that almost half of participants exceeded cutoff scores for PTSD and depression among chemical attack survivors (16, 40, 44).

Our sample also experienced multiple subsequent traumatic events during the chemical attack, with cumulative exposures ranging from 2 to 30 events. Consistent with the ecological model of trauma, prior studies indicate that cumulative exposure to various types of trauma and ongoing environmental stressors is associated with elevated prevalence and severity of PTSD and depression. In this study, we measured cumulative trauma exposure using a validated composite trauma scale. This comprehensive approach probably explains the heightened prevalence and severity of psychological symptoms identified in our sample. These results underscore the complex challenges survivors experience from multiple overlapping stressors, highlighting the need for multi-level mental health interventions (27, 28, 34, 45).

Based on these results, our GLM analysis offers a more nuanced view of how trauma exposure and sociodemographic and clinical factors interact to influence mental health consequences. The GLM analysis shows that psychological symptoms in this population are driven by many interconnected factors, with trauma exposure emerging as a particularly strong predictor across all symptom domains. Consistent gender differences were revealed, with women exhibiting greater vulnerability. Conversely, higher education and income appear as protective factors, particularly regarding somatic symptoms in women, as shown by the interaction between gender and education. Additionally, family psychiatric history, urban residence, and chronic disease were also shown to influence symptom variability, highlighting the intricate interaction of sociodemographic and clinical factors in shaping mental health outcomes. Altogether, our findings underscore the importance of integrated intervention strategies that address trauma, educational inequalities, gender-specific vulnerabilities, socioeconomic support, and broader social factors, in order to reduce long-term psychological distress and promote sustainable improvements in mental health across the Kurdistan region.

Notably, the association between elevated symptoms and psychotropic medication use observed probably indicates practical clinical circumstances where individuals with more severe or treatment-resistant PTSD and depression are predominantly prescribed medication. Meta-analyses reveal modest efficacy of selective serotonin reuptake inhibitors (SSRIs) and related medications, with many individuals showing partial or even no response. Persistent symptoms may arise from complex neurobiological modifications and pharmacological response variability, indicating the limitations of medication alone. These results highlight the importance of tailored treatment strategies that integrate pharmacological, psychotherapeutic, and psychosocial interventions to effectively address the multifaceted needs of trauma survivors (46, 47).

Furthermore, while many participants met cutoff criteria for PTSD, somatic symptoms, and depression/anxiety, fewer reported formal diagnoses or receiving treatment. Specifically, over half had never visited a healthcare provider for mental health concerns, indicating a gap between symptom presence and clinical diagnosis.

Our findings are in line with previous studies that have found that the prevalence of PTSD, depression/anxiety, and somatic symptoms is far more common among women (16, 45–50), especially those who have little or no education (51). In the Kurdish cultural context, women often have limited education and are expected to marry and live as housewives raising children. Lacking employment and experiencing marital conflicts are significant vulnerability factors for developing low self-esteem. This, combined with additional stressful life events, can increase the risk of depression development (52, 53). Importantly, all scales showed good to excellent reliability and strong internal consistency. These findings are consistent with previous studies on these scales in other countries and other samples (37, 54–57). The strong correlations between PCL-5, PHQ-13, and HSCL-25 measures in our study (*r* = 0.69 to 0.80) are consistent with earlier research, confirming the expected overlap and convergent validity of these measures in trauma populations (58).

Although our psychometric measures were robust, the real challenge extends beyond measurement to the lack of adequate services. Despite evidence of a high prevalence of psychological symptoms, people living in the Kurdistan region have very limited or no access to health services in general, let alone psychiatric care, or psychotherapy. The high prevalence of psychopathology and its long-term, chronic effects highlight the urgent need for developing specific treatments and establishing services to support this large group of survivors. Effective treatment strategies are available but have, unfortunately, not yet been implemented in Kurdistan. However, these treatments need to be adapted to the specific local, cultural, and religious needs.

This work uniquely contributes to the literature by employing a multivariate analytical methodology to simultaneously model multiple risk factors, their interaction, and their distinct and combined contributions towards symptom severity. This adds robustness to evidence supporting customized, multidisciplinary intervention approaches encompassing educational support and socio-economically oriented empowerment, especially among susceptible subgroups.

### 4.1 Limitations and future research

In addition to the valuable insights contributed by this study, there are some limitations. First, our sample may be affected by the survivor bias, as a large part of exposed people have died during the elapsed time since the chemical attack. Second, many survivors from Halabja city left the area and now live outside its territory, complicating the sample recruitment process.

Lastly, there is a lack of formal data on psychotherapeutic interventions, further limiting our ability to discern the impact of mental health support on the long-term outcomes. Many participants did not receive structured psychotherapy from trained psychotherapists; instead, they received irregular support and counseling from some organizations that had insufficient resources to address their mental health conditions and may have impacted their recovery.

Future research must investigate, in greater depth, the interaction of psychophysiological, psychological, and socio-demographic (environmental) factors in shaping trauma responses. Future studies should address the limitations related to the generalizability and applicability of our findings.

## 5 Conclusion

This study provides vital evidence on the long-term psychological consequences of chemical weapons exposure, demonstrating the high incidence of PTSD, depression/anxiety, and somatic symptoms among survivors. Our findings show trauma exposure as a strong factor, with sociodemographic factors such as gender, education, and income significantly influencing symptom severity and resilience. Psychometric robustness across multiple validated scales supports the reliability of these insights.

The limited access to mental health services in the Kurdistan region, despite the need, highlights an urgent gap in clinical care and public health infrastructure. These results highlight the urgency for culturally sensitive, multidimensional intervention strategies addressing trauma and its complex socio-environmental correlates.

By combining detailed symptom profiling with multivariate analysis, this study enhances understanding of the long-term psychological effects of trauma and establishes a basis for developing targeted, evidence-based clinical and strategic interventions. Future research should expand on these findings by integrating biological or genetic data to explain trauma mechanisms and enhance intervention approaches.

## Data Availability

The raw data supporting the conclusions of this article will be made available by the authors, without undue reservation.
