# Supplementary material for: A study on long-term trauma-related mental health outcomes among Kurdish survivors of chemical attacks
Source: Front Psychiatry. 2026 Jan 19;16:1693072. doi: 10.3389/fpsyt.2025.1693072 (PMC12862082; doi:10.3389/fpsyt.2025.1693072)
Supplement: Supplementary file 1 [file Table1.docx]

Supplementary Material

**Supplementary Table 1.** Summary of Multivariate General Linear Model Effects of Predictors on Mental Health Outcomes.

The table shows Pillai’s Trace, multivariate F values with degrees of freedom and p-values for predictors showing statistically significant multivariate effects on PHQ13, PCL5, and HSCL25 mental health outcomes.

| Predictor | Pillai’s Trace | Multivariate F (df num, den) | Multivariate p-value |
| --- | --- | --- | --- |
| Gender | 0.187 | 38.66 (3, 504) | < .001 |
| Education | 0.187 | 11.21 (9, 1518) | < .001 |
| Trauma Events | 0.184 | 37.90 (3, 504) | < .001 |
| Family History | 0.038 | 6.69 (3, 504) | < .001 |
| Living Location | 0.049 | 4.25 (6, 1010) | < .001 |
| Chronic Disease | 0.023 | 3.99 (3, 504) | 0.008 |
| Income | 0.205 | 43.41 (3, 504) | < .001 |
| Psychotropic Medication Use | 0.12 | 22.87 (3, 504) | < .001 |
| Prior Diagnosis | 0.086 | 2.485 (18, 1518) | 0.0005 |
