# Supplementary material for: A study on long-term trauma-related mental health outcomes among Kurdish survivors of chemical attacks
Source: Front Psychiatry. 2026 Jan 19;16:1693072. doi: 10.3389/fpsyt.2025.1693072 (PMC12862082; doi:10.3389/fpsyt.2025.1693072)
Supplement: Supplementary file 2 [file Table2.docx]

Supplementary Material

**Supplementary Table 2.** Summary of Univariate General Linear Model Effects of Predictors on Mental Health Outcomes.

*Note:* df = degrees of freedom (numerator, denominator), partial η^2^ = partial eta squared (effect size), β = standardized regression coefficient, and SE = Standard Error.

| Predictor | Outcome | Partial η² | Univariate F (df num, den) | Univariate p-value | β | SE | t-value |
| --- | --- | --- | --- | --- | --- | --- | --- |
| Gender | PHQ13 | 0.18 | 112.64 (1, 506) | < .001 | 0.037 | 1.72 | 0.02 |
| Gender | PCL5 | 0.06 | 30.53 (1, 506) | < .001 | 1.847 | 4.18 | 0.44 |
| Gender | HSCL25 | 0.10 | 54.95 (1, 506) | < .001 | 0.034 | 0.16 | 0.21 |
| Education | PHQ13 | 0.15 | 30.91 (3, 506) | < .001 | -5.17 to -8.38 | 1.52–1.65 | -2.82 to -5.08 |
| Education | PCL5 | 0.09 | 15.70 (3, 506) | < .001 | -7.43 to -12.95 | 3.68–4.01 | -2.02 to -3.23 |
| Education | HSCL25 | 0.12 | 23.31 (3, 506) | < .001 | -0.36 to -0.57 | 0.14–0.16 | -2.24 to -3.70 |
| Trauma Events | PHQ13 | 0.30 | 84.58 (1, 506) | < .001 | 0.337 | 0.06 | 5.76 |
| Trauma Events | PCL5 | 0.38 | 80.43 (1, 506) | < .001 | 0.846 | 0.14 | 5.94 |
| Trauma Events | HSCL25 | 0.34 | 83.82 (1, 506) | < .001 | 0.031 | 0.006 | 5.62 |
| Living Location | PHQ13 | 0.03 | 8.82 (2, 506) | < .001 | 2.54 | 1.05 | 2.42 |
| Living Location | PCL5 | 0.02 | 4.38 (2, 506) | 0.013 | 3.05 | 2.55 | 1.20 |
| Living Location | HSCL25 | 0.04 | 9.44 (2, 506) | < .001 | 0.212 | 0.10 | 2.15 |
| Chronic Disease | PHQ13 | 0.03 | 9.58 (1, 506) | 0.002 | 1.07 | 0.53 | 2.00 |
| Income | PHQ13 | 0.46 | 125.75 (1, 506) | < .001 | -0.0093 | 0.0009 | -10.12 |
| Income | PCL5 | 0.34 | 51.46 (1, 506) | < .001 | -0.012 | 0.002 | -5.60 |
| Income | HSCL25 | 0.57 | 68.15 (1, 506) | < .001 | -0.00056 | 0.00009 | -6.47 |
| Psychotropic Medication Use | PHQ13 | 0.05 | 25.74 (1, 506) | < .001 | 2.31 | 1.00 | 2.32 |
| Psychotropic Medication Use | PCL5 | 0.08 | 42.89 (1, 506) | < .001 | 6.93 | 2.42 | 2.86 |
| Psychotropic Medication Use | HSCL25 | 0.12 | 67.43 (1, 506) | < .001 | 0.30 | 0.09 | 3.25 |
